# Macro-Meso Characteristics and Damage Mechanism of Cement-Stabilized Macadam Under Freeze–Thaw Cycles and Scouring

**DOI:** 10.3390/ma18214874

**Published:** 2025-10-24

**Authors:** Hongfu Liu, Sirui Zhou, Ao Kuang, Dongzhao Jin, Xinghai Peng, Songtao Lv

**Affiliations:** 1 National Engineering Research Center of Highway Maintenance Technology, Changsha University of Science and Technology, Changsha 410114, China; lst@csust.edu.cn; 2 School of Transportation, Changsha University of Science and Technology, Changsha 410114, China; 23101030124@csust.edu.cn; 3 Guangdong Jianke Traffic Engineering Quality Inspection Center Co., Guangzhou 510599, China; 17673763151@163.com; 4 Deparment of Civil and Environmental Engineering, Michigan Technological University, 1400 Townsend Dr., Houghton, MI 49931, USA; 5 Institute of Highway Engineering, RWTH Aachen University, 52074 Aachen, Germany; peng@isac.rwth-aachen.de

**Keywords:** cement-stabilized macadam, acoustic emission, mesoscopic pore characteristics, gray correlation analysis, damage mechanism

## Abstract

This study quantifies the effects of freeze–thaw (FT) cycling and dynamic water scouring, and establishes links between mesoscale pore evolution and macroscale strength degradation in cement-stabilized macadam (CSM) bases. The objective is to provide quantitative indicators for durability design and non-destructive evaluation of CSM bases. First, laboratory tests were conducted to simulate alpine service conditions: CSM cylindrical specimens (Ø150 × 150 mm) with 4.5% cement content, cured for 28 days, were exposed to 0, 5, or 20 FT cycles (−18 °C for 16 h ↔ +25 °C for 8 h), followed by dynamic water scouring (0.5 MPa, 10 Hz) for 15, 30, or 60 min. Second, the resulting damage was tracked at two scales. Acoustic emission (AE) sensors monitored internal damage during subsequent splitting tests, while industrial computed tomography (CT) was used to scan selected specimens and quantify porosity, pore number, and average pore diameter. Third, gray relational analysis correlated pore structure parameters with strength loss. The results indicate that under 30 min of scouring, increasing FT cycles from 0 to 20 increased mass loss from 0.33% to 1.27% and reduced splitting strength by 28.8%. AE cumulative ringing count and energy decreased by 97.9% and 98.4%, respectively, indicating severe internal degradation. CT scans revealed porosity and pore count increased monotonically with FT cycles, while average pore diameter decreased (dominated by microcrack formation). Frost-heave pressure and cyclic suction enlarged edge pores and interconnected internal voids, accelerating erosion of cement paste. FT cycles compromise the cement–aggregate interfacial bond, thereby predisposing the matrix to accelerated deterioration under dynamic scouring; the ensuing evolution of pore structure emerges as the pivotal mechanism governing strength degradation. Average pore diameter exhibited the strongest correlation with splitting strength (r = 0.763), and its change was the primary driver of strength loss (r = 0.774). These findings facilitate optimizing cement dosage, validating non-destructive evaluation models for in-service base courses, and erosion durability of road base materials in permafrost regions.

## 1. Introduction

By the end of 2024, China’s total road network had reached 5.49 million km, with a specific highway mileage of 190,700 km [1]. China has built the largest and most diverse highway transportation system in the world. Owing to the complexity of construction conditions and the wide variation in service environments, particularly in severe climates such as the Qinghai–Tibet Plateau, China faces uniquely high demands for pavement durability [2]. Among the various structural forms, semi-rigid-base asphalt pavement structure is the main structural form of highway and urban road construction in China. It benefits from its high bearing capacity, good durability, wide source, and low price. Cement-stabilized macadam (CSM) is a composite material formed by water and cementitious materials uniformly coated on the surface of different-particle-size minerals after a hydration reaction. Currently, it is extensively utilized in the semi-rigid bases of high-grade highways in China, offering the benefits of readily available materials and straightforward construction techniques [3].

CSM exhibits favorable strength and stability, but it remains highly susceptible to environmental damage [4,5]. In particularly frigid areas like the Qinghai–Tibet Plateau in China, the significant temperature fluctuations and atypical weather patterns of heavy precipitation and snowfall create a challenging environment for roads, resulting in semi-rigid-base pavements exhibiting pores and varying degrees of cracking. Once the surface layer fractures, rainwater will persist in infiltrating the base layer, resulting in localized humidity and a tendency towards saturation in the base layer. The water is stranded inside the base layer and cannot be effectively discharged. Under cyclic temperature variations, the pavement base endures alternating freeze–thaw (FT) effects from free water, which makes the FT cycle of the road severe and further aggravates the water damage. Simultaneously, under the action of traffic load, the static water produces downward instantaneous pressure when it is squeezed. When multiple vehicles pass by, it causes repeated compression and suction processes. The water is repeatedly washed in the gap of the base material, gradually eroding the cement and resulting in a decrease in the adhesion of the aggregate. With the repeated accumulation of damage under the action of high-water pressure, the cracks develop rapidly, which further leads to the damage of CSM, such as squirt mud, mud pumping, and voiding, and causes damage to the pavement. Under the synergistic effects of numerous FT cycles and dynamic water scouring, CSM deteriorates, leading to strength loss in the road base, reduced load-bearing capacity, and consequent decline in pavement serviceability [6,7,8].

Given the harsh environmental conditions and the associated road damage in severely cold regions, researchers have studied the properties of CSM materials [9], mix proportions [10], forming method [11], and service environment [12]. By optimizing the design of pavement structure layer and adopting multi-index control, the strength and modulus of the base layer are in a reasonable range under specific environmental conditions, and its performance is improved [13]. A large number of scholars have conducted extensive research according to the actual environmental conditions of the materials. Most studies focus on climatic impacts on pavement performance in cold regions, assessing frost resistance and thermal shrinkage characteristics of CSM, while proposing various evaluation indices to quantify material frost resistance [14,15,16].

Meanwhile, engineering applications in permafrost regions must account for the synergistic effects of FT cycles and dynamic scouring on CSM. To reduce the occurrence of damage to CSM bases, researchers from multiple nations have examined the frost and erosion resistances of CSM from various perspectives. The research on frost resistance mainly focuses on factors such as cement dosage, flexural strength, number of FT cycles, and early strength agent [17]. It has made substantial breakthroughs and provided valuable research basis and empirical basis for subsequent research. It has been determined that maintaining the cement percentage between 4% and 6% optimizes the material’s frost resistance. Nevertheless, when surpassing the threshold, the proliferation of microcracks induced by the elevation of hydration heat increases the occurrence of FT degradation. By establishing a unified strength degradation calculation model and frost resistance evaluation index, the influence of cement content and fine particle removal size on the FT mechanical performance of CSM was quantitatively characterized [18]. The effect of the FT cycle affects the flexural strength and fatigue performance of the CSM mixture. During FT cycle tests, the extent of fatigue damage diminishes rapidly in the initial phase and gradually in the subsequent phase. FT damage predominantly transpires at the periphery and primary void [19,20]. The frequent freezing and thawing of semi-rigid base materials in high-altitude regions negatively impacts their performance. Researchers have evaluated the frost resistance of semi-rigid base materials in high-altitude areas by establishing correlations between FT cycle numbers and both compressive and splitting frost resistance coefficients [21]. Incorporation of the early-strength agent decreases material water absorption and mass loss, with the frost resistance coefficient progressively increasing at higher agent contents. Song et al. examined the impact of various dosages of early strength agents on the frost resistance of CSM materials and determined the optimal additive content [22].

However, the research on macro performance is difficult to fully evaluate the FT damage of the base and propose a reasonable control method. Numerous studies have investigated the FT damage mechanisms in semi-rigid base materials and have proposed several notable theories regarding FT damage based on investigations in the realm of concrete materials. Xie et al. categorized the damage process into four stages: initial damage prior to the FT cycle, water absorption saturation, damage progression, and complete damage, and formulated a parabolic model of FT damage to forecast the service life of materials [23]. Combined with fracture mechanics, Li et al. proposed a mesoscale model for FT cracking that considers both pore structure alterations and cyclic damage effects. The new model can more accurately assess the crack breadth of concrete resulting from FT cycles [24]. Zhao et al. examined the damage constitutive behavior under coupled FT cycling and loading conditions, formulating a constitutive model that simulates the complete deformation response of CSM materials subjected to both FT actions and uniaxial compression [25]. Li et al. examined the skeletal structure characteristics of CSM from a microscopic perspective, analyzed the effects of various molding techniques and gradations on this skeletal structure, and introduced a novel evaluation index for the skeletal structure of CSM [26].

Road researchers conducted a series of tests to evaluate the anti-scouring effectiveness of semi-rigid foundation materials. Guo et al. demonstrated that the scour resistance of CSM improves with higher cement content, extended curing age, and increased strength [27]. Yu et al. made a new scouring test device and carried out a dynamic water pressure scouring test on the CSM material. The apparent morphology and strength of the specimens before and after scouring were analyzed, and the scouring failure mechanism of the CSM base was studied [28]. Liu conducted an in-depth analysis of the scouring and fatigue properties of the base material, assessed the primary elements influencing the anti-scouring efficacy of the CSM base, and employed the fatigue damage theory to examine the fatigue deterioration of the base post-scouring [29]. Sheng et al. examined the elements influencing the anti-erosion efficacy of semi-rigid base materials, established the design index for the composition of these materials, and introduced a design methodology for semi-rigid base material composition predicated on anti-erosion performance [30]. Some existing methods simulate the scouring process of the base to a certain extent. Nevertheless, there is a paucity of research about the failure mode of the semi-rigid base under dynamic water pressure, and the evolution process of the surface behavior of the specimen under the action of water flow is not considered [31,32].

Although the existing studies have discussed the frost resistance and erosion resistance of CSM, most of them focus on performance changes under the action of a single factor, lack of systematic analysis of FT, and scouring coupling effect, and there is a certain degree of difference between the study of damage mechanism and the study of various properties. Therefore, for a long time, there has been a large deviation between the study of the damage state of pavement base materials and structures and the damage state under actual conditions. Researchers have failed to combine the actual environment of the base material and comprehensively consider the damage caused by various factors to the material. At the same time, the impact of various features is predominantly analyzed from a macro viewpoint, while research on the material’s damage mechanism from a mesoscopic perspective is comparatively limited. It is difficult to comprehensively analyze the failure mechanism of CSM semi-rigid base materials and propose reasonable disposal methods. Consequently, this study aims to systematically investigate the coupled effects of freeze–thaw cycles and dynamic water scouring on the mechanical degradation and mesoscopic damage evolution of cement-stabilized macadam (CSM). It is essential to conduct a focused study based on its material characteristics and actual service conditions, and it is practically significant to investigate the mechanical properties and damage mechanisms of CSM under FT and scouring conditions.

Overall, the existing studies investigating the effects of freeze–thaw cycles or scouring on cement-stabilized macadam (CSM) still have three key limitations:

(1) Most tests focus on single-factor conditions, lacking coupled freeze–thaw–scour simulations that fail to replicate the actual service environment in cold plateau regions.
(2) The existing damage assessments rely primarily on macroscopic strength and mass loss measurements, with no established quantitative correlation between pore evolution and mechanical degradation.
(3) The detailed process of crack initiation, propagation, and penetration is insufficiently characterized, and sensitive indicators suitable for on-site non-destructive testing are lacking.

To address these limitations, this study proposes an integrated research framework of “coupled environment–macroscopic/mesoscopic damage–quantitative indicators”:

(1) Laboratory simulations replicate the coupling of freeze–thaw cycles and dynamic water scouring, with systematic collection of mass loss, splitting strength, and full-process acoustic emission data.
(2) Using CT scanning, porosity, pore number, and average pore diameter are extracted; combined with gray correlation analysis, average pore diameter is identified as the dominant factor governing mechanical property degradation.
(3) A damage evaluation index based on pore evolution is developed, providing quantitative support for durability design, non-destructive testing, and maintenance decision-making of semi-rigid bases in cold regions.

**Figure 1:**
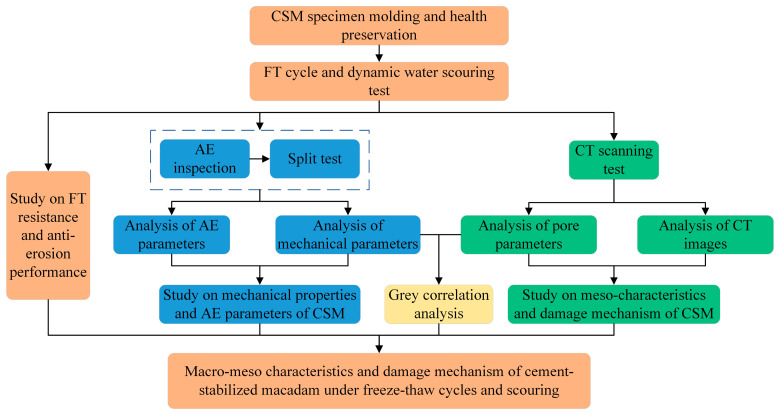
The research framework diagram.

The technology roadmap of this paper is shown in Figure 1. The study provides critical theoretical support for both understanding FT damage behavior and designing countermeasures in cement-stabilized base layers under harsh cold climates, particularly in high-altitude areas such as the Tibetan Plateau.

## 2. Materials and Methods

### 2.1. Materials and Mix Design

**Figure 2:**
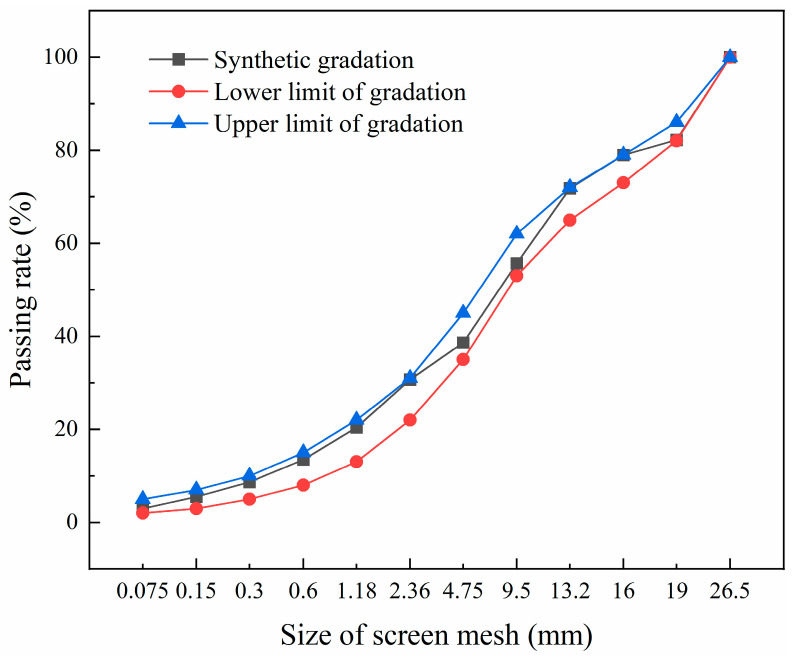
Grading curve of cement-stabilized macadam material.

The aggregates used in this study were sourced from Zhongshan quarry, with a maximum particle size of 26.5 mm. Following the gradation requirements of CSM in the Technical Guidelines for Construction of Highway Roadbases (JTG/T F20-2015) [33] and Specifications for Design of Highway Asphalt Pavement (JTG D50-2017) [34], the aggregate of each grade was screened, and the gradation curve is shown in Figure 2. The study utilized ordinary Portland cement (P.O 42.5), and the suspended dense structure was selected. The CSM mixture was mixed with 4.5% cement content and a water–cement ratio of 1.0, exhibiting a maximum dry density of 2.351 g/cm^3^ and an optimum moisture content of 4.5%.

According to the Test Methods of Materials Stabilized with Inorganic Binders for Highway Engineering (JTG 3441-2024) [35], the mass of each aggregate fraction required for the specimen was calculated from the maximum dry density and optimum moisture content. The batched aggregates were first moistened by adding water, sealed in a container, and left to equilibrate. A predetermined mass of cement was weighed and mixed with water, after which the cement was added to the pre-saturated mixture and homogenized thoroughly. The mixture was then added into the test mold in three increments, with simultaneous compaction performed using a vibrator during each addition. After all the mixture was placed and rodded completely, CSM cylinder specimens with a size of Ø150 mm × 150 mm were prepared by the vibration molding method. The specimens were bagged one by one and placed in a standard curing room at 20 ± 2 °C for 28 days. The research programs are shown in Table 1.

### 2.2. Test Method

#### 2.2.1. Test of FT Cycle and Dynamic Water Scouring

The average yearly frequency of FT cycles in the alpine region is predominantly 120–150 times, and the number of indoor equivalent FT cycles is more than 14 times [36]. In accordance with JTG 3441-2024 [35], the tests of FT cycle and dynamic water scouring were carried out on the CSM specimens with FT cycles of 0, 5, and 20 times and scouring times of 15 min, 30 min, and 60 min, respectively, to simulate the evolution of material properties under different service environments. The process is as follows:

(1) The specimens after peeling and curing were immersed in water for 24 h.
(2) The specimen was removed from the water, the water on its surface was wiped off, and the quality of the cylinder specimen was weighed.
(3) The specimens were subjected to FT cycle tests with different FT cycles. After weighing, the specimens were quickly placed in an FT test chamber. The basic parameters of the FT test were as follows: the freezing control temperature was −18 ± 2 °C, and the freezing time was 16 h. After the freezing was completed, the specimens were taken out and ablated in a room with a constant temperature of 25 ± 1 °C, and the melting time was 8 h.
(4) The MTS scouring platform was used to conduct scouring tests on the specimens under different scouring conditions after the FT cycles. The impact force was determined to be 0.5 MPa; the loading frequency was 10 Hz; and the scouring time was 15 min, 30 min, and 60 min. The anti-erosion performance of the CSM specimens was studied. After the scouring was completed, the specimens in the scouring bucket were taken out, and the quality of the specimens after the scouring was weighed.
(5) The aforementioned testing procedure was repeated, and the test specimen was subjected to the FT cycle test and the scour test individually.

#### 2.2.2. Splitting Acoustic Emission Test

**Figure 3:**
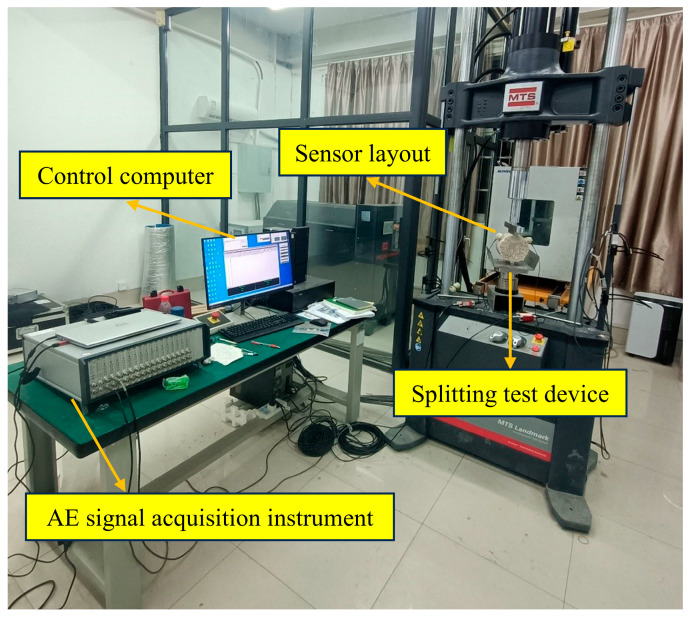
AE system detection process.

Figure 3 illustrates the acoustic emission detection system used for the specimen. Sensors were configured in a dual-channel arrangement to capture acoustic emission signals during the specimen’s splitting failure [37,38].

Upon selecting the appropriate channel in the signal processing software, the acoustic emission intensity threshold was established at 40 dB, with signals beyond this threshold being captured, and the preamplifier was calibrated accordingly. The filter range was established at 25 kHz to 850 kHz, with a sampling frequency of 10 MHz. To prevent the sensor from detaching and compromising data collection, a layer of Vaseline was applied to the surface prior to positioning the sensor, ensuring a superior fit between the sensor and the specimen.

The MTS universal testing machine (Land-Mark, MTS Systems, Eden Prairie, MN, USA) was used to test the splitting strength of the specimen [39]. Simultaneously, the acoustic emission acquisition system was initiated to document the stress state and acoustic emission signal parameters of the specimen. The test was halted upon the specimen attaining the maximal stress state.

#### 2.2.3. CT Scanning Test

**Figure 4:**
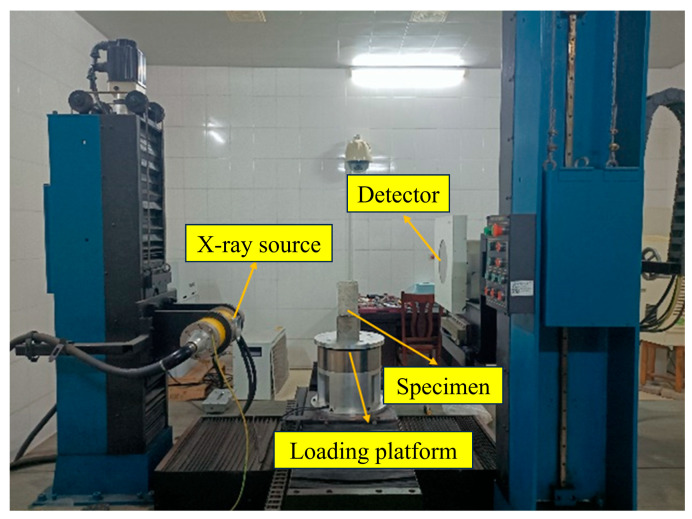
Industrial CT scanning equipment.

Industrial CT scanning (MG325, YXLON, Hamburg, Germany) was performed on the CSM specimens subjected to 0, 5, or 20 FT cycles followed by 30 min of dynamic water scouring. The pore structure distribution of each section was obtained, and the changes in the pores were compared and analyzed. Excessive size compromises the resolution quality of scanned CT images [40]. Therefore, a cylindrical specimen of Ø100 mm × 150 mm was first drilled by core sampling. A total of six specimens were numbered 1, 2, 3, 4, 5, and 6. The specimens 1, 2, and 3 were the test groups with 5 FT cycles, and the specimens 4, 5, and 6 were the test groups with 20 FT cycles. The specimens were scanned before freezing, thawing, and scouring. The sections of 40 mm, 50 mm, 60 mm, 70 mm, 80 mm, 90 mm, 100 mm, 110 mm, and 120 mm from the bottom of the specimen were analyzed. The industrial CT scanning equipment is shown in Figure 4.

## 3. Results and Discussion

### 3.1. Analysis of FT and Scouring Resistance

#### 3.1.1. Analysis of Mass Loss Rate

**Figure 5:**
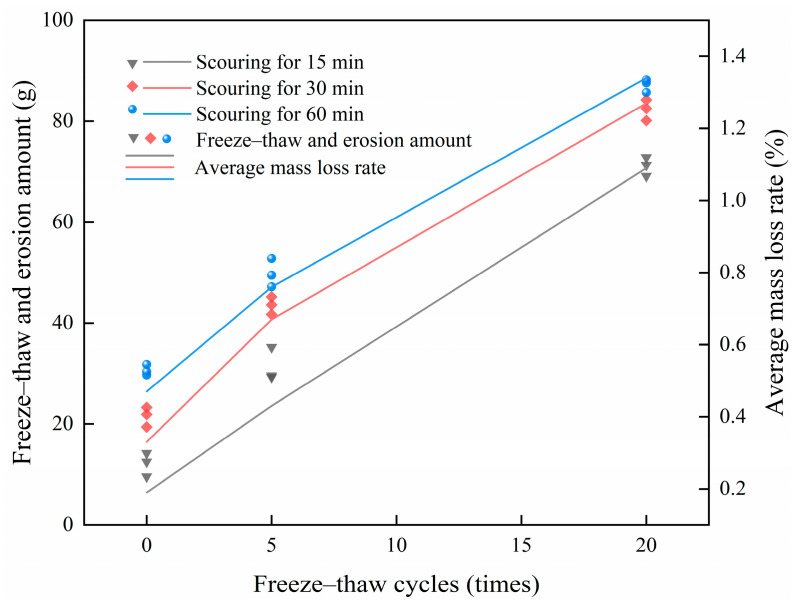
Average mass loss rate of specimens under different FT washout conditions.

In this paper, the mass loss rate is used to analyze the FT and scouring resistance of CSM. The scouring mass loss rate of the specimens after the test is calculated according to Equation (1). The test results are shown in Table 2 and Figure 5.

(1) $$S=\frac{m_{f}-m_{0}}{m_{0}}$$

where S is the scouring mass loss rate (%); $m_{f}$ is the quality of specimen after scouring (g); and $m_{0}$ is the initial mass of specimen (g).

The test results demonstrate that the mass loss rate of CSM exhibits a positive correlation with the scouring duration under identical FT cycles. Under sustained dynamic water pressure, the adhesive force between the cement hydration products and aggregates deteriorates, causing progressive spalling of cement paste from the specimen. At the same time, the aggregate becomes loose under the continuous action of the upper load. With the increase in scouring time, the amount of FT and scouring of CSM increases. Under equivalent numbers of FT cycles, the average mass loss rate increment of the scouring time from 15 min to 30 min is larger than that from 30 min to 60 min, which indicates that the mass loss of the CSM material becomes slower after 30 min of scouring. The mass loss of the specimen is mainly caused by the shedding of cement hydrates and fine materials in the base material. The incremental mass loss of the material after 30 min scouring shows a decreasing trend, as prolonged scouring duration progressively removes the weakly adhered fine particles from the CSM, resulting in diminishing scouring losses.

With a scouring duration of 30 min, the mass loss rate of the specimen escalates from 0.33% to 1.27% as the number of FT cycles rises from 0 to 20. The CSM specimens exposed to 5 and 20 FT cycles demonstrate markedly elevated FT and scouring amount, and mass loss rates, in comparison to the non-cycled specimens, with these effects intensifying as cycle numbers increase. The FT cycle reduces the bonding force between the cement slurry and the aggregate of the specimen, so that the scouring effect is more obvious. The combined effect of the FT cycle and scour aggravates the loss of the specimen and increases the degree of damage. The experimental data reveal that the damage caused by FT to the specimen structure leads to an increase in the amount of scouring in the scouring test. The main reason is that the FT cycle leads to the damage of the cohesive force between the cement hydrate and the aggregate inside the specimen, which leads to the aggregate and the cement hydrate being more likely to be washed away when the material is washed by dynamic water, thereby increasing the amount of scouring.

#### 3.1.2. Analysis of Splitting Strength

**Figure 6:**
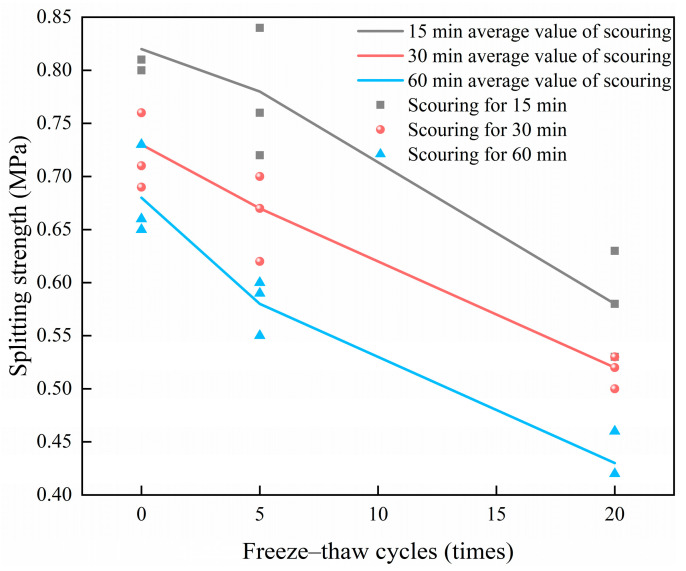
Splitting strength test results of specimens under different FT scour conditions.

Table 3 and Figure 6 present the splitting tensile strength test results of the CSM specimens under varying FT cycles and dynamic water scouring conditions, showing the variation law of the splitting strength of the specimens affected by the number of FT cycles and scouring time.

Figure 6 illustrates that with consistent scouring duration, the splitting strength of the specimen diminishes progressively as the number of FT cycles increases. With a scouring duration of 30 min, the splitting strength diminishes by 28.8% as the number of FT cycles escalates from 0 to 20. Under the same FT conditions, the splitting strength shows a consistent downward trend with the increase in scouring time. The splitting strength of CSM decreases greatly after 15 min to 30 min of dynamic water scouring, while the splitting strength decreases slightly after 30 min to 60 min of dynamic water scouring. Because the CSM specimen mainly acts on the side surface of the specimen when it is scoured, with the increase in the scouring time, the duration of the dynamic water pressure increases. The FT cycle reduces the adhesion between the side aggregate and the cement, and the surface aggregate and cement are washed away by water under the condition of dynamic water scouring. At the same time, large pores are generated, which further aggravates the damage of the material and leads to a decrease in the splitting strength of the CSM specimen.

### 3.2. Analysis of Acoustic Emission Parameters

According to the analysis of the splitting test results, it can be seen that the mechanical properties of CSM have different degrees of change under the influence of FT cycle and dynamic water scouring. To investigate the failure process of CSM, acoustic emission (AE) parameters recorded during splitting tests were employed to characterize internal damage progression in the specimens subjected to identical scouring duration (30 min) but varying FT cycles (0, 5, and 20 times). The number and strength of the AE activities are reflected by a comprehensive analysis of load, time, AE amplitude, ringing count, and energy, and these parameters are used to describe the damage state of different specimens during splitting loading.

#### 3.2.1. Amplitude Analysis

**Figure 7:**
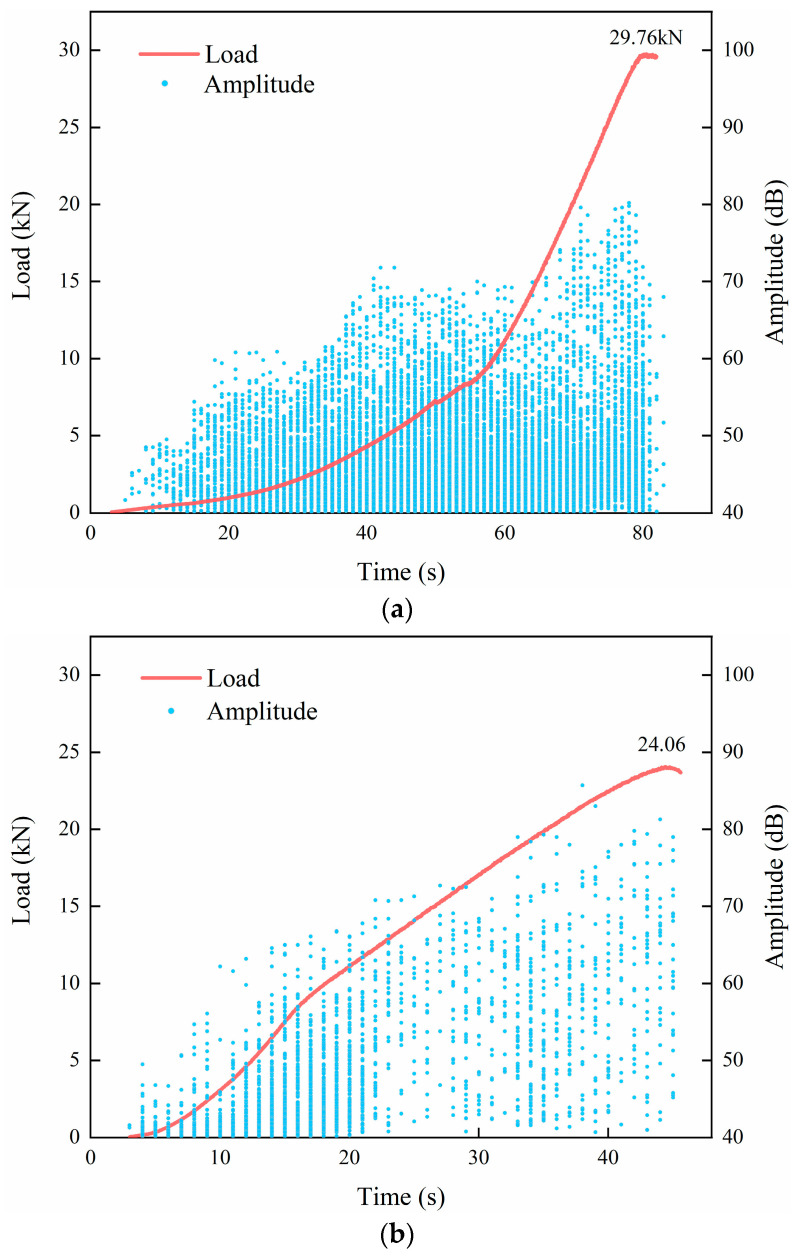

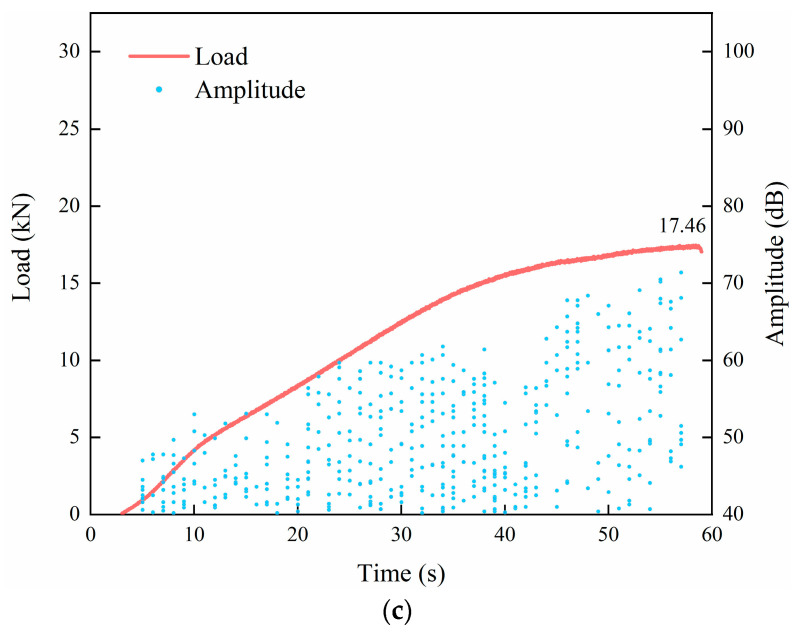
Plot of acoustic emission amplitude and load–time relationship of cement-stabilized macadam under splitting action: (**a**) 0 F-T cycles; (**b**) 5 F-T cycles; (**c**) 20 F-T cycles.

The relationship between the AE amplitude and the load level of CSM under the splitting condition with time is shown in Figure 7. According to the parameter changes in the AE signal in Figure 7, the splitting failure process of the CSM specimen can be divided into three stages: the initial damage stage of the specimen, the internal damage development stage of the specimen, and the damage failure stage of the specimen.

As shown in Figure 7, analysis of the load–time curves reveals 19.15% and 41.33% reductions in peak load for specimens subjected to 5 and 20 FT cycles, respectively, relative to non-cycled controls, demonstrating a progressive decline in peak strength with increasing FT exposure. The loading time of the specimens after the FT treatment to reach the peak load is shorter than that of the specimens without the FT cycle, indicating that the splitting strength of the CSM specimens after the FT cycle has a certain degree of deterioration, and the specimens have certain damage.

The amplitude change in the AE signal of CSM is divided into three stages. The first stage is the initial damage stage of the specimen. The load continuously pressurizes the specimen. At this time, the load is relatively small. The amplitude of the AE signal is mainly distributed between 40 dB and 55 dB, and the number of larger AE amplitudes is relatively small. This indicates that during initial loading stages, due to the effect of splitting, the CSM specimen is in a compacted state, and the initial damage is less.

The second stage is the internal damage development stage of the specimen. As the load increases, the distribution of the amplitude of the AE signal diverges. The amplitude range of the second stage is larger than that of the first stage, mainly distributed between 40 dB and 70 dB. With the increase in splitting load, the internal damage of CSM specimens is in the development stage, the microcracks expand, and more cracks appear in the specimens, but the overall increase is relatively stable.

The third stage represents the specimen’s damage and failure phase. As the load increases to the peak value, the amplitude of the AE signal is further increased to a high value, with a range of more than 70 dB. This is because the internal damage of the specimen continues to occur under the splitting load, and the damage gradually develops into macroscopic cracks.

Under the same scouring conditions, the AE signals of the CSM specimens with different FT cycles are compared. The frequency of acoustic emission events diminishes progressively with an increase in the FT cycles, signifying that these cycles significantly affect the specimens, leading to a marked deterioration in their attributes. As a result of the FT cycle, the specimen’s capacity to endure splitting loads diminishes progressively, leading to the formation of microcracks and other forms of damage, thereby weakening the detected AE signal.

#### 3.2.2. Ringing Count and Energy Analysis

**Figure 8:**
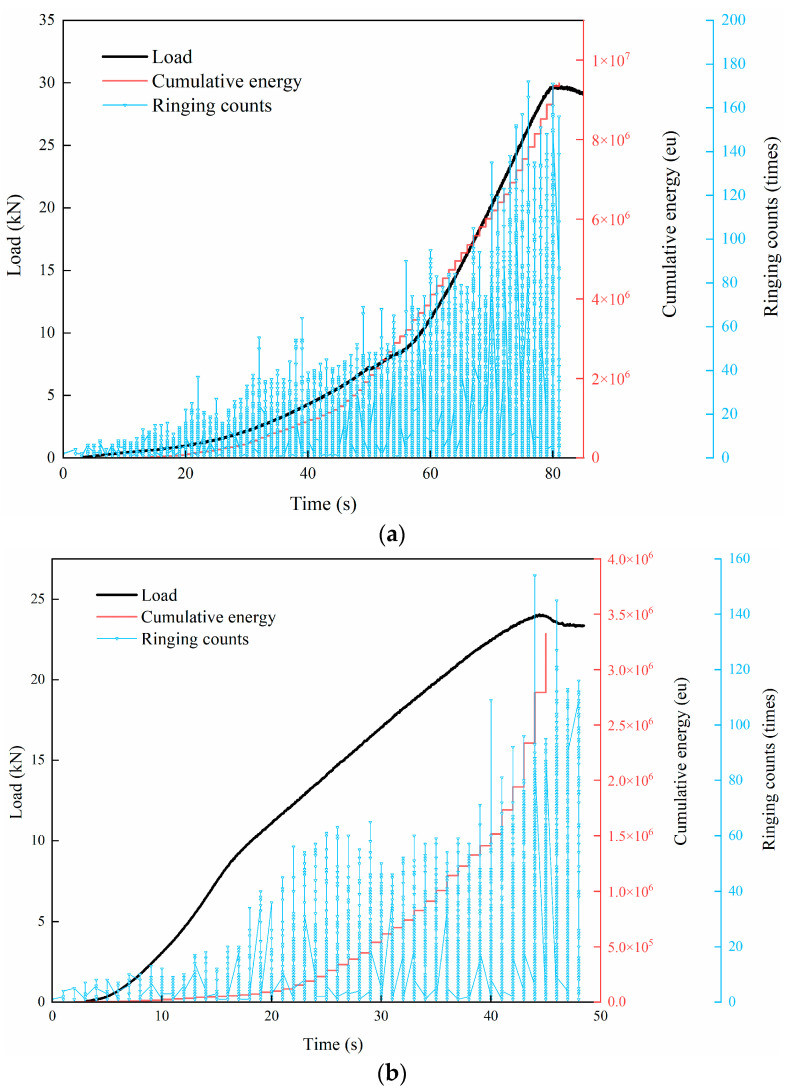

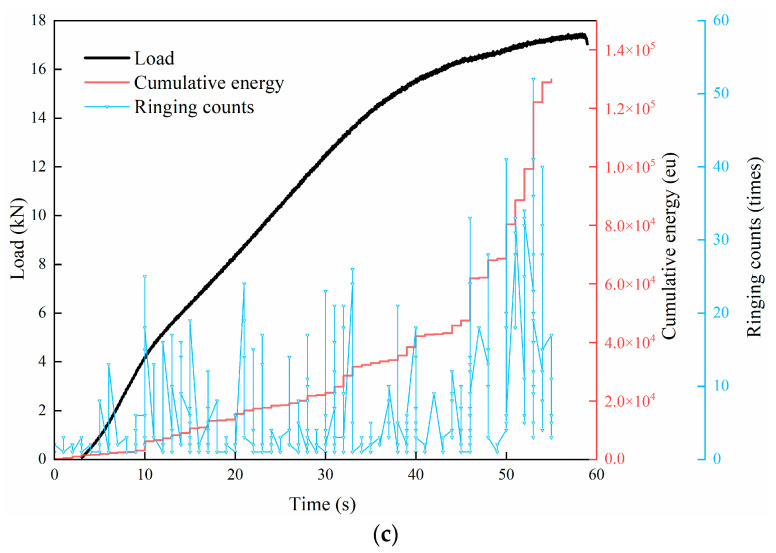
Plot of ringing counts, cumulative energy, and load–time relationship of cement-stabilized macadam under splitting action: (**a**) 0 F-T cycles; (**b**) 5 F-T cycles; (**c**) 20 F-T cycles.

This section analyzes the extent of damage to the specimen by examining the variations in ringing count and cumulative energy during the splitting failure process of the CSM specimen. Table 4 presents the AE ringing count and cumulative energy data for CSM subjected to various FT and scouring conditions. The relationship between the AE parameters and load level with time is shown in Figure 8.

Analysis of Table 4 data, combined with the variations in ringing count and cumulative energy depicted in Figure 8, reveals that the increase in AE ringing count and energy values recorded during the splitting process of specimens devoid of FT cycles is relatively stable. As the load increases, both the ringing count and the cumulative energy curve rise steadily. As the number of FT cycles escalated, the ringing count and cumulative energy value of the specimen increased rapidly after loading for a period of time, showing a jump growth and obvious stage characteristics. The mean cumulative AE ring counts and cumulative energy recorded during the specimen’s splitting process, absent an FT cycle, reached 185,697 eu and 8,627,507 eu, respectively. The mean cumulative AE ring counts and cumulative energy recorded during the specimen’s splitting process, subjected to 5 FT cycles, reached 108,790 eu and 4,042,385 eu, respectively, representing reductions of 41.4% and 50.1% compared to the specimen without FT cycles. The mean cumulative AE ring counts and mean cumulative energy diminished by 41.4% and 50.1%, respectively. After 20 cycles of freezing and thawing, the average cumulative AE ring counts and cumulative energy collected during the specimen’s splitting process were 3943 times and 138,620 eu, respectively. These values represent reductions of 97.9% and 98.4%, respectively, compared with those of the unfrozen and thawed specimens. This shows that compared with the specimens without FT and scouring, the CSM specimens with FT and scouring have less AE signal activity, and the cumulative AE ringing count and cumulative energy parameters have been greatly reduced, which also shows that different FT and scouring conditions have different damage degrees to CSM.

The three stages of the splitting failure process were analyzed. In the initial damage stage of the specimen, the AE ringing count is lower and its value is lower. The cumulative energy value increases relatively slowly, and the variation characteristics of the two are similar, indicating that the AE activity generated in this stage is low, and the intensity of the AE signal is low. During the first stage of loading, the pores at the internal stress of the specimen are compacted under the action of the splitting load to produce a small amount of microcrack damage and a small amount of AE signal, and no damage can be seen on the surface of the specimen. As FT cycles escalate, the growth trends of AE ringing counts and energy stay comparable during this period. Nevertheless, both the ringing count and energy values decline progressively, accompanied by a decrease in the AE event frequency and signal intensity. This shows that the FT damage to the performance and internal structure of the material specimens has a greater growth trend.

The specimen then entered the internal damage development stage. Compared with the first stage, with the continuous improvement of the load level, the ringing count and density began to increase, and the cumulative energy increased steadily at this stage. Under the action of splitting load, the cracks in the specimen continue to develop, the degree of internal damage continues to increase, and more cracks appear, but the overall increasing trend is relatively stable.

The third stage is the damage and failure stage of the specimen. During this stage, the load action duration is brief, the AE activity is heightened, the strength of the AE signal is substantial, and both the ringing count and cumulative energy escalate rapidly. This indicates that the specimen has a sudden brittle failure at this stage, and the internal microcracks develop into macroscopic cracks that can be observed on the surface. The FT damage has the greatest influence on the damage stage of the specimen. The greater the degree of freezing and thawing of the specimen, the more serious the deterioration of its own properties and the faster the damage, so the AE signal parameters in the collected failure process change faster.

### 3.3. Mesoscopic Characteristics and Damage Mechanism Analysis

Based on the analysis and research on the failure process of CSM, in order to explore the essential mechanism of material damage, this paper explores the damage mechanism and internal pore structure changes in CSM material under FT and scouring from a mesoscopic perspective. The internal pore characteristics of the specimen were obtained by CT technology, and the damage mechanism of the material was analyzed by the obtained CT image information, which further provided the basis for the damage characteristics of CSM.

#### 3.3.1. Analysis of CT Images and Cumulative Damage Mechanism of FT and Scouring

**Figure 9:**
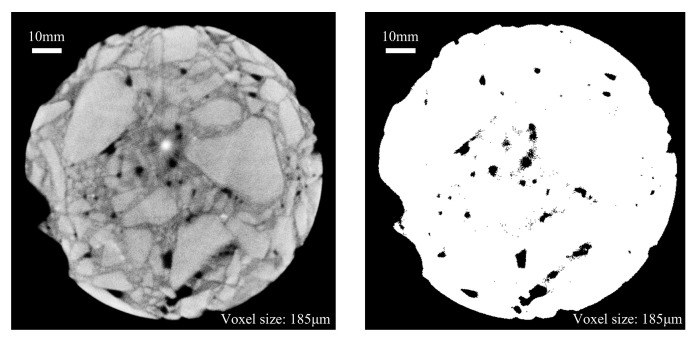
CT images before and after image processing.

The specimens subjected to the FT cycle and dynamic water scouring test were scanned using CT, yielding XY-plane CT scan pictures of two groups: one with 5 FT cycles and another with 20 FT cycles, to analyze the alterations in internal pore structure. The software Image-Pro Plus 6.0 was used to preprocess the cross-section CT images obtained by scanning, enhance the brightness and contrast of the whole image, remove the ring shadow of the image, and improve the light and dark contrast relationship of aggregates and pores. Then, the image segmentation technology was used to segment the pore structure, and the gray threshold was adjusted to separate the aggregate, cement mortar, and pore. The pore part in the image was marked as black by adjusting the threshold, and the rest of the aggregate and cement were adjusted to white. Finally, a binary CT image that makes it easy to identify the pore structure was obtained. The comparison between the CT images before and after processing is shown in Figure 9.

Three sections were selected in the upper, middle, and lower parts of the specimen in turn, and the corresponding pore distribution diagram was obtained as follows. The black part in the figure is the pore of the specimen.

**Figure 10:**
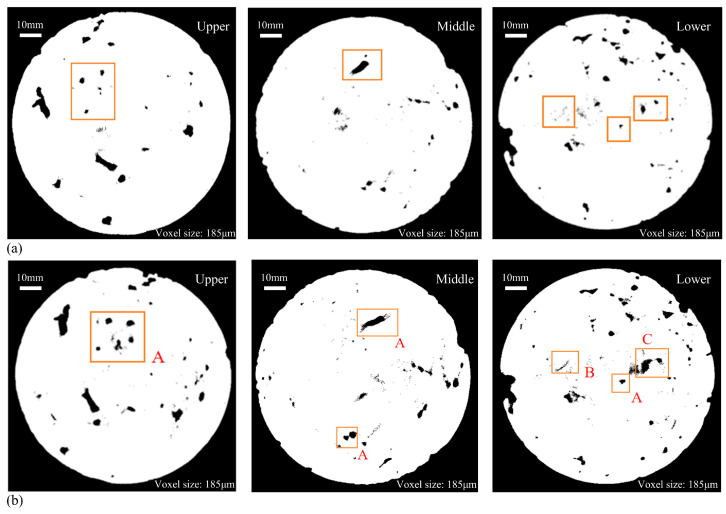
CT scan pictures (5 FT cycles): (**a**) before FT and scouring; (**b**) after FT and scouring.

**Figure 11:**
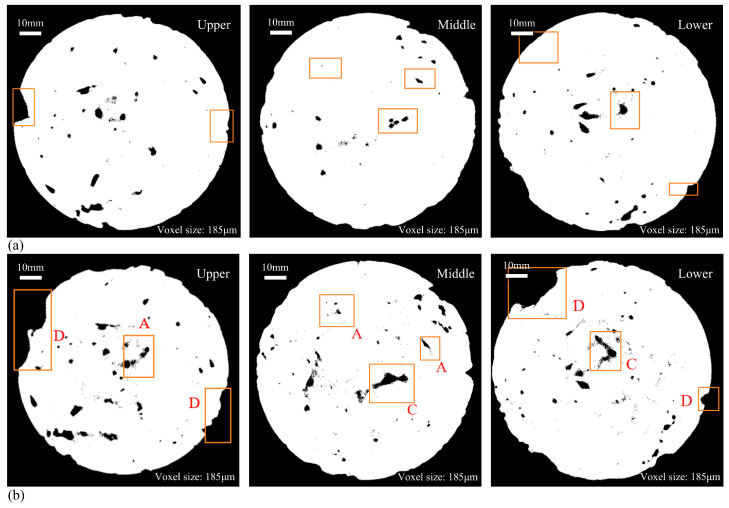
CT scan pictures (20 FT cycles): (**a**) before FT and scouring; (**b**) after FT and scouring.

The cross-section CT images of the upper, middle, and lower parts of the specimens in different FT cycle test groups under the condition of scouring for 30 min are shown in Figure 10 and Figure 11. The information represented by the position and letters selected in the box in the image is as follows:

A: New pores and expanded original pores.

B: Cracks formed inside the specimen.

C: The connection between pores leads to the change in pore structure in the specimen.

D: The position where the cement and aggregate on the outer surface of the specimen peeled off from the specimen.

It can be seen from the CT section map after the binarization treatment that the surface deterioration of the specimen is obvious after 30 min of scouring, the internal pores change to varying degrees, and the number and area of pores increase significantly. The pores increased by the FT cycle are mostly generated at the edge of the specimen, and the expansion of the original pores in the interior. At the same time, there are also some new micro-pores, such as the A-type markers in the figure. The voids generated during the FT process of CSM materials are positively correlated with the number of original pores. The damage degree of the upper and lower sections of the specimen is more serious than that of the middle part, and the pore area is obviously larger. This is due to the uneven mixing of cement and coarse and fine aggregates, and the vibration molding process. FT and scouring make the outer surface of the specimen more vulnerable to damage.

Pores are present on the specimen’s edge prior to the FT cycle, and subsequent to the cycle, surface cement mortar spalling occurs at the same location on the specimen’s outer edge, leading to enlarged pores. Comparing the CT images of 5 FT cycles and 20 FT cycles, it is evident that as the number of FT cycles increases, the frost heave degree of water on the edge of the specimen becomes more serious, and the peeling of cement and aggregate becomes more serious. The new opening gap and the shedding of large aggregates in the peripheral part of the specimen become more serious (as shown in the D mark in Figure 11), resulting in an increase in porosity, which is caused by the gradual penetration of cracks from the surface to the interior of the specimen.

The internal pore structure and distribution of the specimens changed to a certain extent after the FT cycles and dynamic water scouring. In the low-temperature environment, the water inside the base specimen gradually froze, and the resulting frost heave force caused the closed pores inside the CSM specimen to frost heave and stretch, and caused the specimen to crack. With the increase in temperature, the ice in the pores gradually melted into liquid water. At the same time, the specimen continued to bear the load, which made the liquid water pressure in the closed pores change dramatically, and the positive pressure and negative suction accelerated the crack propagation of the specimen. Under the alternating action of dynamic water scouring and FT cycle, the volume of closed pores in CSM specimens increased continuously, which reversely aggravated the frost heaving force in the FT process and the scouring effect of flowing water in the ablation process. In addition, due to the combined effect of FT cycles and dynamic water scouring, the pores were connected with each other (as shown in the C mark in Figure 11), resulting in a substantial enlargement of the initial pores and exacerbating cracking. By analyzing the D mark position in Figure 11, it can be seen that there are no pores at the edge of the specimen before freezing and thawing, while after 20 FT cycles, the outer surface of the specimen has visible large aggregate shedding and new opening voids. This is due to the effect of FT cycles. The new pores inside the specimen cause damage to the specimen. At the same time, the cumulative effect of dynamic water scouring causes the frost heave pores of the specimen and the shedding and stripping of the edge aggregate to be more significant, and the pore volume further increases, resulting in damage to the specimen.

#### 3.3.2. Parameter Analysis of Mesoscopic Pore Structure Change

The Image-Pro Plus software was used to binarize the CT cross-section image to obtain the internal structure information of the CSM, and the porosity, pore number, and average pore diameter of the test piece were calculated to more fully study the FT cycle and dynamic water scouring’s influence on the microstructure of CSM. By analyzing the changes in pore parameters, the formation and development of internal damage of CSM were analyzed.

##### Porosity

**Figure 12:**
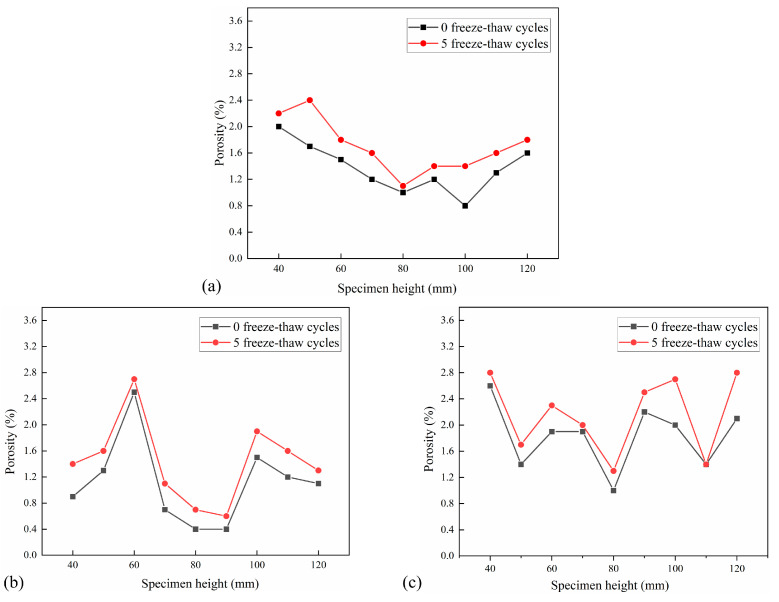
Porosity distribution after 5 FT cycles: (**a**) No. 1 specimen; (**b**) No. 2 specimen; (**c**) No. 3 specimen.

**Figure 13:**
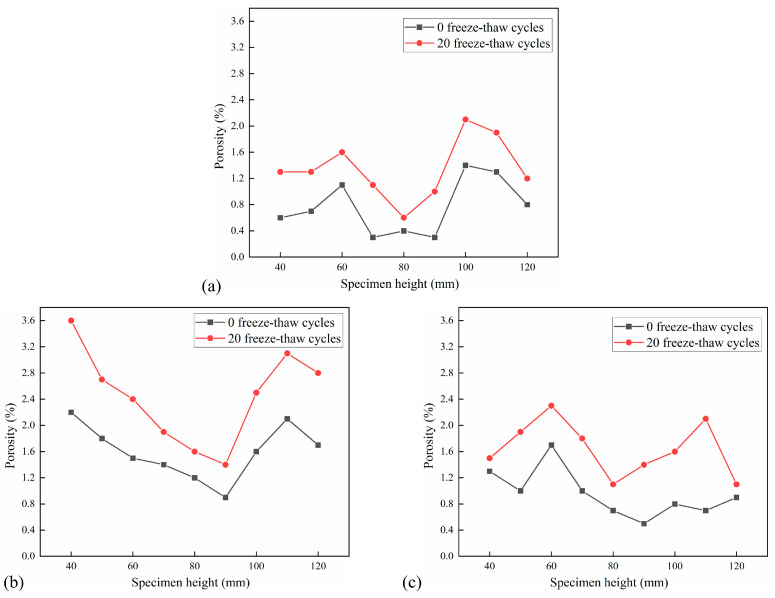
Porosity distribution after 20 FT cycles: (**a**) No. 4 specimen; (**b**) No. 5 specimen; (**c**) No. 6 specimen.

The cross-sectional porosity distribution of each interval of 10 mm in the height of CSM specimens under 5 FT cycles and 20 FT cycles is shown in Figure 12 and Figure 13. Each test group has three parallel specimens. The porosity increment under different F-T cycles is shown in Table 5.

Figure 12 and Figure 13 illustrate that the porosity distribution among various specimens is analogous, exhibiting a general pattern of high porosity at both extremities and low porosity in the center. Concurrently, porosity in CSM exhibits a positive correlation with FT cycles. Table 5 illustrates that the cross-sectional porosity increment of the CSM specimens with 5 FT cycles is 0–0.7%, and the average porosity increment is 0.33%. The cross-sectional porosity increment of the CSM specimens after 20 FT cycles is 0.2–1.4%, with an average porosity increment of 0.70%, indicating that increasing FT cycles significantly affects the internal porosity of CSM. The effect of dynamic water scouring in the FT cycles makes the internal pores of the specimen gradually expand and develop, and at the same time, new pores are generated inside the specimen, which leads to an increase in porosity, so that the internal stress of the material is concentrated, which accelerates the expansion of microcracks, thereby reducing the splitting strength of the material, and ultimately leads to the continuous development and deterioration of the internal damage of the CSM specimen.

##### Pore Number

**Figure 14:**
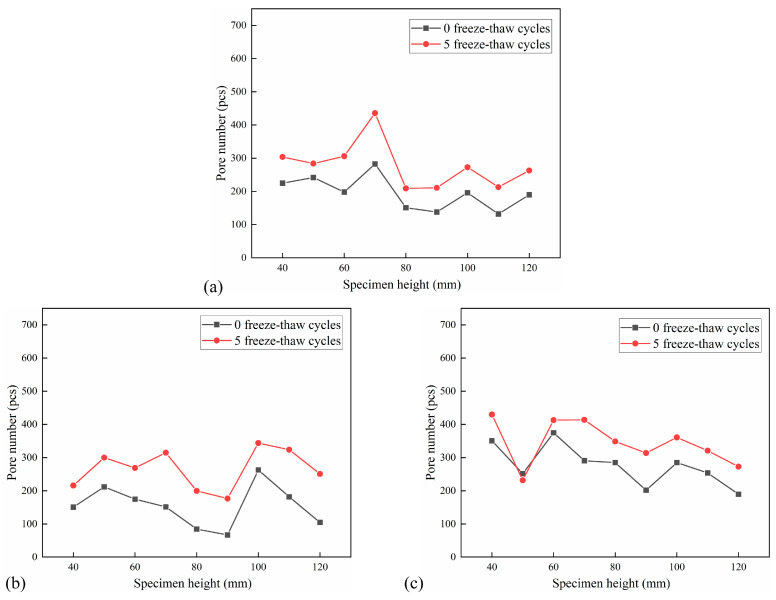
Pore number distribution after 5 FT cycles: (**a**) No. 1 specimen; (**b**) No. 2 specimen; (**c**) No. 3 specimen.

**Figure 15:**
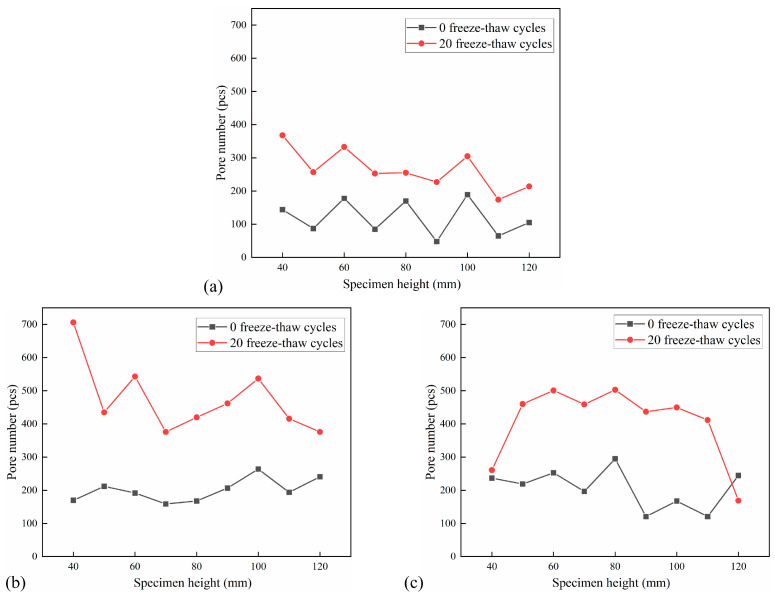
Pore number distribution after 20 FT cycles: (**a**) No. 4 specimen; (**b**) No. 5 specimen; (**c**) No. 6 specimen.

The distribution of the number of pores per 10 mm in the height of the CSM specimen under the action of 5 FT cycles and 20 FT cycles is shown in Figure 14 and Figure 15. The pore number increment under different F-T cycles is shown in Table 6.

The analysis of pore number in the cross-section of each specimen indicates that the pore number in the CSM specimen escalates with an increasing number of FT cycles. The number of holes in the CSM specimens that have undergone 20 FT cycles increased significantly. Due to the increase in the number of FT cycles, the water migrated to the interior of the specimen, and the periodic frost heave effect caused damage to the interior of the specimen, which made the CSM specimen produce new pores, and the existing pores gradually expanded and developed, and the number of pores inside the material increased rapidly. Furthermore, the pore data indicates that when the number of FT cycles increases, certain sections exhibit a trend of pore reduction. This is due to the effect of FT cycles. Although new pores are generated inside the specimen, the pores are developed into larger connected pores due to the effect of dynamic water scouring, and the number of pores has a decreasing trend. The change in the number of pores also shows that the damage to the CSM specimens is getting worse.

##### Average Pore Diameter

**Figure 16:**
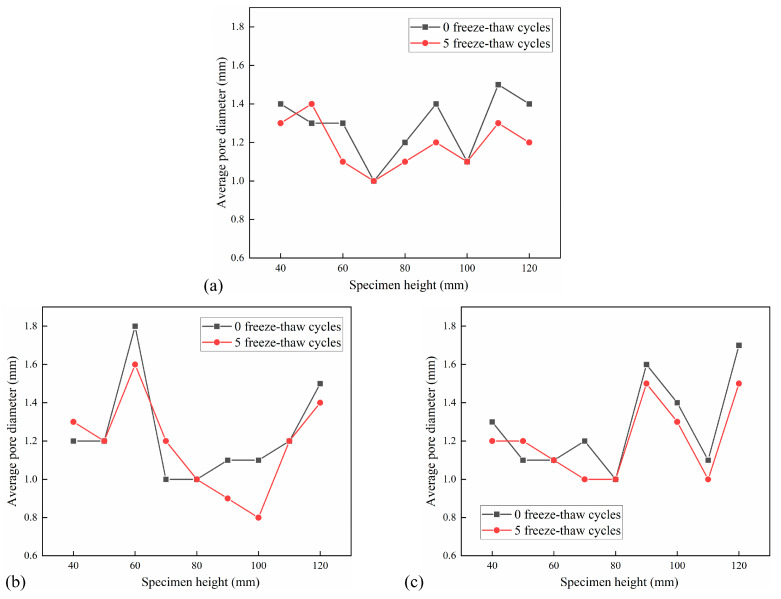
Distribution of average pore diameter in 5 FT cycles: (**a**) No. 1 specimen; (**b**) No. 2 specimen; (**c**) No. 3 specimen.

**Figure 17:**
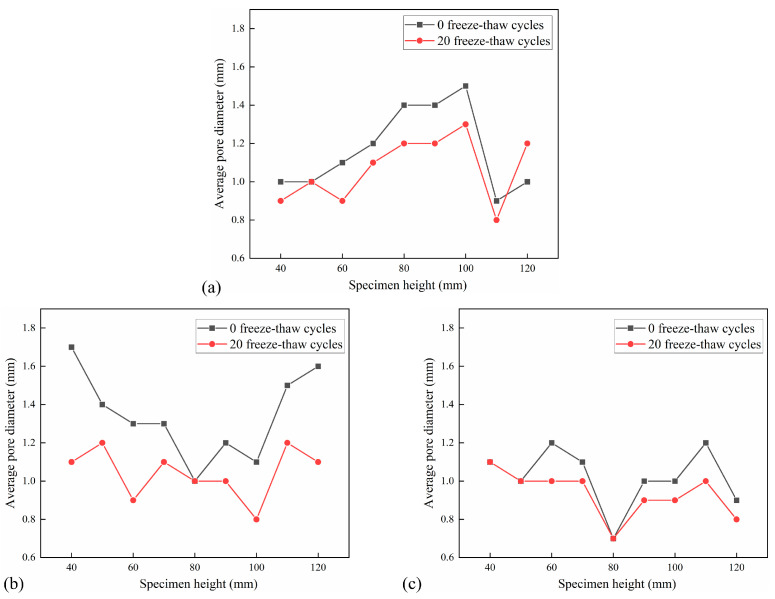
Distribution of average pore diameter in 20 FT cycles: (**a**) No. 4 specimen; (**b**) No. 5 specimen; (**c**) No. 6 specimen.

The distribution of the average pore diameter of the cross-section of the CSM specimen with an interval of 10 mm under the action of 5 FT cycles and 20 FT cycles is shown in Figure 16 and Figure 17. The average pore diameter decrement under different F-T cycles is shown in Table 7.

It can be observed from Figure 16 and Figure 17 that the average pore diameter of the specimen decreases with the increase in the number of FT cycles. This is because the FT damage inside the specimen leads to an increase in new pores and an increase in the number of tiny pores. According to Table 7, the average pore diameter of the cross-section of the test group with 5 FT cycles decreased by 0.06–0.10 mm, while the average pore diameter of the cross-section of the test group with 20 FT cycles decreased by 0.10–0.29 mm, indicating that the more FT cycles, the more new micro-pores. The average pore diameter of a small part of the cross-section increases, which is due to the effect of the FT cycle and dynamic water scouring, which makes the pores develop into larger connected pores, resulting in an increase in the average pore diameter of the pores.

#### 3.3.3. Correlation Analysis of Macro and Micro Parameters

The internal structure of CSM material determines its macroscopic mechanical properties. The effect of FT cycle and dynamic water scouring leads to the deterioration of the internal pore structure of the CSM specimen, which causes the specimen to be damaged and the mechanical properties to decrease. Therefore, based on the gray correlation theory [41], this section studies the correlation between the macroscopic mechanical properties and the mesoscopic pore structure parameters of CSM under the action of FT cycles and dynamic water scouring.

The cross-sectional porosity, pore number, and average pore diameter obtained by scanning the specimen without the action of the FT cycle–dynamic water scouring condition were used as the comparison sequence $X_{i}$, and the splitting strength was used as the reference sequence $X_{0}$ to calculate the gray correlation degree.

Table 8 presents the original data of pore characteristic parameters and compressive strength of specimens without FT cycle and dynamic water scouring. Based on the gray correlation theory, the correlation between different pore parameters and compressive strength was analyzed.

The correlation between splitting strength and pore structure parameters is calculated as follows: $r_{1}=0.514$, $r_{2}=0.618$, and $r_{3}=0.763$.

$r_{1}$, $r_{2}$, and $r_{3}$ are the correlation degrees between porosity, pore number, average pore diameter, and splitting strength, respectively; $r_{3}>r_{2}>r_{1}$.

In summary, the order of the gray correlation degree between the pore parameters and splitting strength of CSM without FT and scouring is as follows: average pore diameter > pore number > porosity.

In order to clarify the main factors of the influence of the change in pore characteristic parameters on the mechanical properties of CSM under the action of FT cycle and dynamic water scouring, the porosity, pore number, and average pore diameter of the specimens after FT cycle and dynamic water scouring were scanned. The change value was used as a comparison sequence, and the loss value of splitting strength was used as a reference sequence. The correlation between different pore parameters and splitting strength loss was calculated. Table 9 presents the original data of pore structure parameters variation value and splitting strength loss value.

**Figure 18:**
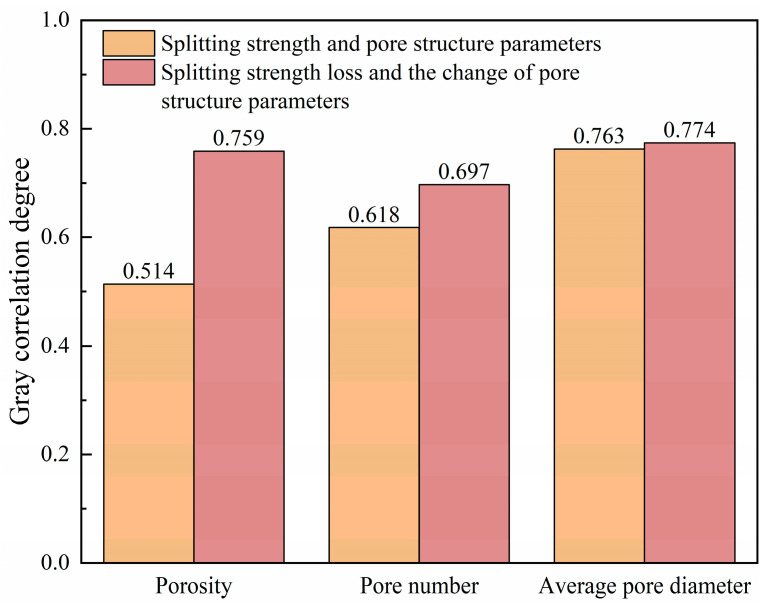
Gray correlation degree calculation results.

The correlation between the splitting strength loss and the change in pore structure parameters is calculated as follows: $r_{1}=0.759$, $r_{2}=0.697$, and $r_{3}=0.774$. The gray correlation degree calculation results are shown in Figure 18.

$r_{1}$, $r_{2}$ and $r_{3}$ are the correlation degrees between porosity, pore number, average pore diameter changes, and splitting strength loss, respectively; $r_{3}>r_{1}>r_{2}$.

In summary, the gray correlation degree between the change value of porosity, pore number, average pore diameter, and the loss value of splitting strength of the specimens after the FT cycle and dynamic water scouring is as follows: average pore diameter > porosity > pore number.

Compared with the above correlation calculation results, it can be seen that the correlation between the average pore diameter and the splitting strength is the highest in the pore characteristic parameters of CSM without the FT cycle and dynamic water scouring. After the FT cycle and dynamic water scouring, the change in average pore diameter has the greatest correlation with the loss of splitting strength, which indicates that the change in average pore diameter is the main factor leading to the attenuation of the mechanical properties of CSM. The change in porosity has little effect, while the change in pore number has the least effect on mechanical properties. The correlation conclusion can provide quantitative indicators for non-destructive testing technology and improve the FT resistance and anti-scouring durability of road base in severe cold areas.

## 4. Conclusions

This study investigates the macro–micro mechanical behavior and damage evolution mechanisms of cement-stabilized macadam under FT cycle and dynamic water scouring. The FT resistance and scouring durability of CSM were evaluated through laboratory FT cycling tests, dynamic water scouring testing, splitting strength measurements, acoustic emission monitoring, and CT scanning. The failure process of the specimen was characterized by acoustic emission parameters, and the mesoscopic pore characteristics and damage mechanism of different CSM specimens were analyzed from a mesoscopic perspective. Based on the gray correlation theory, the correlation analysis of macro and micro parameters was carried out. The main conclusions are as follows:

(1) With a scouring duration of 30 min and an increase in FT cycles from 0 to 20, the mass loss rate of the specimen escalated from 0.33% to 1.27%, while the splitting strength diminished by 28.8%. The FT cycle aggravated the damage to the specimen, which was a significant factor in the reduction in strength.
(2) With the increase in FT cycles from 0 to 20, the cumulative ringing count of CSM specimens decreased from 185,697 times to 3943 times, the cumulative energy decreased from 8,627,507 eu to 138,620 eu, and the peak load decreased by 41.33%. The acoustic emission parameters diverged and decreased significantly with the increase in the number of FT cycles, indicating that the damage of the specimen was more serious.
(3) CT scans revealed monotonic increases in porosity and pore count with FT cycles, whereas average pore diameter decreased (indicating dominant microcracking). The volume of pores in the specimens was escalating, exacerbating the frost heaving force during the FT cycle and the erosive impact of running water in the scouring process. Frost-heave pressure, followed by suction during thawing/scouring cycles, progressively interconnected isolated pores, leading to macro-crack formation. Water infiltrated the fractures and generated a suction effect during the FT cycle, resulting in crack propagation and exacerbating the damage to the specimens.
(4) The gray correlation results show that the pore structure parameters of the specimens had a good correlation with their mechanical properties. The correlation degree between average pore diameter and splitting strength was the largest, and the correlation degree was r = 0.763. After the specimen was subjected to FT cycle and dynamic water scouring, the correlation between the change value of the average pore diameter and the loss value of the splitting strength was the largest, and the correlation degree was r = 0.774, indicating that the change was the dominant factor in the deterioration of mechanical properties. The correlation conclusion can provide quantitative indicators for non-destructive testing technology.

This study solely employed cross-sectional CT images to investigate the internal pore distribution and structural changes in CSM specimens, with the limitation of a small sample size used for CT scanning. In subsequent work, it is recommended to expand the sample size and reconstruct three-dimensional images of CSM base materials from CT scans. This will enable precise quantification of the 3D pore volume, pore count, and aggregate distribution, thereby facilitating a more in-depth analysis of the damage characteristics of CSM. Moreover, expanding the sample size and reconstructing 3D models from CT data will not only allow for accurate quantification of pore volume, count, and aggregate distribution but also provide high-fidelity 3D models that can serve as direct inputs for numerical finite element analysis, enabling comparative studies between experimental observations and finite element simulations.

## Figures and Tables

**Table 1:** Research programs.

Batch ID	Specimen Composition	Curing Age/d	Specimen Size/mm	No. of Specimens	Test Type
A-1	CSM-4.5%C	28	Ø150 mm × 150 mm	27	FT cycle and dynamic water scouring
A-2	CSM-4.5%C	28	Ø150 mm × 150 mm	30	Splitting acoustic emission test
A-3	CSM-4.5%C	28	Ø100 mm × 150 mm	6	CT scanning test

**Table 2:** Freeze–thaw and erosion amount and mass loss rate of CSM under different conditions.

Scouring Time	F-T Times (Times)	Freeze–Thaw and Erosion Amount (g)				Average Mass Loss Rate (%)
		Specimen 1	Specimen 2	Specimen 3	Average	
15	0	9.6	12.5	14.2	12.1	0.19
	5	35.2	29.6	29.3	30.0	0.43
	20	71.3	72.8	69.1	71.1	1.09
30	0	23.3	21.9	19.4	21.5	0.33
	5	43.6	41.7	45.2	43.5	0.67
	20	82.5	84.2	80.1	82.3	1.27
60	0	29.7	30.4	31.8	30.6	0.47
	5	49.5	47.2	52.8	51.5	0.76
	20	87.6	88.2	85.7	87.2	1.34

**Table 3:** Table of splitting strength test results of specimens under different FT scour conditions.

Scouring Time	F-T Times (Times)	Splitting Strength (MPa)			
		Specimen 1	Specimen 2	Specimen 3	Average
—	—	0.84	0.96	0.87	0.90
15	0	0.80	0.86	0.81	0.83
	5	0.72	0.84	0.76	0.78
	20	0.58	0.63	0.53	0.58
30	0	0.71	0.76	0.69	0.73
	5	0.67	0.62	0.70	0.67
	20	0.53	0.50	0.52	0.52
60	0	0.73	0.65	0.66	0.68
	5	0.55	0.59	0.60	0.58
	20	0.46	0.42	0.42	0.43

**Table 4:** AE cumulative ringing count and cumulative energy data.

Scouring Time	F-T Times (Times)	Cumulative Ringing Count (Times)	Mean Cumulative Ringing Count (Times)	Cumulative Energy (eu)	Average Cumulative Energy (eu)
30 min	0	208,218	185,697	9,425,708	8,627,507
		163,176		7,829,306	
	5	97,264	108,790	4,758,276	4,042,385
		120,315		3,326,493	
	20	2237	3943	129,911	138,620
		5648		147,328	

**Table 5:** Porosity increment under different F-T cycles.

F-T Times (Times)	The Average Porosity Increment (%)				Maximum Porosity Increment (%)	Minimum Porosity Increment (%)
	Specimen 1	Specimen 2	Specimen 3	Average		
5	0.33	0.32	0.33	0.33	0.7	0
20	0.58	0.84	0.69	0.70	1.4	0.2

**Table 6:** Pore number increment under different F-T cycles.

F-T Times (Times)	The Average Pore Number Increment (pcs)			
	Specimen 1	Specimen 2	Specimen 3	Average
5	83	111	69	88
20	146	274	201	207

**Table 7:** Average pore diameter decrement under different F-T cycles.

F-T Times (Times)	Average Pore Diameter Decrement (mm)			
	Specimen 1	Specimen 2	Specimen 3	Average
5	0.10	0.06	0.07	0.08
20	0.10	0.29	0.11	0.17

**Table 8:** Pore structure parameters and splitting strength raw data.

Serial Number	$\mathrm{Splitting}\;\mathrm{Strength}/(\mathrm{MPa})\;X_{0}$	Porosity/ $(\%)\;X_{1}$	$\mathrm{Pore}\;\mathrm{Number}/(\mathrm{Pieces})\;X_{2}$	$\mathrm{Average}\;\mathrm{Pore}\;\mathrm{Diameter}/(\mathrm{mm})\;X_{3}$
1	0.84	1.367	1755	1.289
2	0.96	1.100	1392	1.233
3	0.87	1.833	2485	1.279
4	0.80	0.767	1071	1.167
5	0.86	1.600	1807	1.344
6	0.81	0.956	1856	1.022

**Table 9:** Pore structure parameters variation value and splitting strength loss value raw data.

Serial Number	$\mathrm{Splitting}\;\mathrm{Strength}\;\mathrm{Loss}\;\mathrm{Value}/(\mathrm{MPa})\;X_{0}$	Porosity/ $(\%)\;X_{1}$	$\mathrm{Pore}\;\mathrm{Number}/(\mathrm{Pieces})\;X_{2}$	$\mathrm{Average}\;\mathrm{Pore}\;\mathrm{Diameter}/(\mathrm{mm})\;X_{3}$
1	0.42	0.333	744	0.089
2	0.46	0.432	1004	0.055
3	0.42	0.334	622	0.079
4	0.53	0.578	1315	0.100
5	0.50	0.844	2464	0.300
6	0.52	0.688	1796	0.089

## Data Availability

The original contributions presented in this study are included in the article. Further inquiries can be directed to the corresponding authors.

## References

[1] Statistical Bulletin of Transportation Industry Development in 2024. https://www.gov.cn/lianbo/bumen/202506/content_7027415.htm.

[2] Editorial Department of China Journal of Highway and Transport (2024). Review on China’s Pavement Engineering Research: 2024. China J. Highw. Transp..

[3] Shen W., Zheng X., Liu H., Xu J., Wang Q., Zhao D. (2021). Review on Classification and Service Status of Road Base Course. J. Wuhan Univ. Technol..

[4] Ma S., Gao J., Wei J., Zhang X. (2019). Study on Unconfined Compressive Strength and Frost Resistance of Low Temperature Early Strength Cement Stabilized Macadam Material. Bull. Chin. Ceram. Soc..

[5] Zhang M., Fang Z., Liu H., Jiao S. (2016). The Pavement Performance of Cement Stabilized Crushed Stones and Corresponding Research on Adaptation of Heavy Traffic. J. Shenyang Jianzhu Univ..

[6] Chen J., Zhang M., Wang H., Li L. (2015). Evaluation of Thermal Conductivity of Asphalt Concrete with Heterogeneous Microstructure. Appl. Therm. Eng..

[7] Dongzhao J., Wang J., You L., Ge D., Liu C., Liu H., You Z. (2021). Waste cathode-ray-tube glass powder modified asphalt materials: Preparation and characterization. J. Clean. Prod..

[8] Dongzhao J., Meyer T.K., Chen S., Boateng K.A., Pearce J.M., You Z. (2022). Evaluation of lab performance of stamp sand and acrylonitrile styrene acrylate waste composites without asphalt as road surface materials. Constr. Build. Mater..

[9] Mandal T., Edil T.B., Tinjum J.M. (2018). Study on Flexural Strength, Modulus, and Fatigue Cracking of Cementitiously Stabilised Materials. Road Mater. Pavement Des..

[10] Liu Z., Liu J., Wang Q., Liu J. (2015). Compressive Strength and Frost Heave Resistance of Different Types of Semi-Rigid Base Materials After Freeze-Thaw Cycles. Sci. Cold Arid Reg..

[11] Liu L., Wang C., Liang Q., Chen F., Zhou X. (2023). A State-of-the-Art Review of Rubber Modified Cement-Based Materials: Cement Stabilized Base. J. Clean. Prod..

[12] Jiang Y., Zhang Y., Yi Y., Fan J., Tian T. (2024). Performance Degradation Rules of Cement-stabilized Macadam with Construction Waste Recycled Aggregate Under Environmental Loads. J. Highw. Transp. Res. Dev..

[13] Li X., Zhou Y., Xu J., Ning Z., Dia A. (2023). Macro- and microscale properties of vibration mixing cement-stabilized macadam: Laboratory and field investigation. Iran. J. Sci. Technol. Trans. Civ. Eng..

[14] Zhang Y., Wang A., Zhang J., Ma T., Chen S. (2023). Dry Shrinkage in Cement-Stabilized Macadam: A Review. J. Jilin Univ..

[15] Su P., Liu Y., Li M., He Z., You Z. (2022). Simulation on Strength and Thermal Shrinkage Property Mechanisms of Pre-Cracked Cement Stabilized Crushed Stone. J. Traffic Transp. Eng..

[16] Lin M., Han H., Dong X., Song Y. (2017). Experiment Study of Early Strength Agent on Dry Shrinkage Characteristic of Cement Stabilized Macadam Material. Bull. Chin. Ceram. Soc..

[17] Tang H., Yang Y., Li H., Xiao L., Ge Y. (2023). Effects of chloride salt erosion and freeze–thaw cycle on interface shear behavior between ordinary concrete and self-compacting concrete. Structures.

[18] Jie S., Yue Z., Sun Z., Zhang H., Hu T., Sun T., Yuan L., Xie P., Mao S. (2022). Frost Resistance and Mechanical Degradation Characteristics of Cement-Stabilized Macadam Under the Long-Term Freezing–Thawing Cycles. Constr. Build. Mater..

[19] Wang Y., Tan Y., Wang X. (2017). Study on Fatigue Characteristics of Freeze-Thaw Damage of Cement Stabilized Macadam. Highway.

[20] Wang Y., Tan Y., Guo M., Wang X. (2017). Effect of Freeze-Thaw Cycles on the Internal Structure and Performance of Semirigid Base Materials. Adv. Mater. Sci. Eng..

[21] Tian Y., Ma B., Wang D., Li N. (2017). Freeze Resistance Characteristics of Cement-Stabilized Macadam Under Freeze-Thaw Cycle. J. Chang’an Univ..

[22] Song Y., Li L., Liu Y., Lin M. (2016). Influence of Early Strength Agent on Frost Resistance Performance of Cement Stabilized Material. J. Highway Transp. Res. Dev..

[23] Xie C., Cao M., Yin H., Guan J., Wang L. (2021). Effects of Freeze-Thaw Damage on Fracture Properties and Microstructure of Hybrid Fibers Reinforced Cementitious Composites Containing Calcium Carbonate Whisker. Constr. Build. Mater..

[24] Li B., Mao J., Shen W., Liu H., Liu X., Xu G. (2019). Mesoscopic Cracking Model of Cement-Based Materials Subjected to Freeze-Thaw Cycles. Constr. Build. Mater..

[25] Zhao L., Yan Z., Xu S., Ren S., Wang Y., Chi L. (2025). Mesoscopic Damage Mechanism of Multiple Freeze–Thaw Cycles of Cement Gravel Based on Particle Flow Theory. Comp. Part. Mech..

[26] Li Z., Liu Y., Liu J. (2023). Research on Characteristics of Meso-Skeleton Structure of Cement-Stabilized Rock Material. J. Henan Polytech. Univ..

[27] Guo R., Nian T., Li P., Fu J., Guo H. (2019). Anti-Erosion Performance of Asphalt Pavement with a Sub-Base of Cement-Treated Mixtures. Constr. Build. Mater..

[28] Yu H., Zhao Q., Zhang C., Feng B. (2019). Experimental Study of Dynamic Water Erosion to Cement Stabilized Macadam Base. Highway.

[29] Liu J., Wang S., Shi K., Zhang C. (2022). Anti-erosion Performance and Fatigue Characteristics of Cement Concrete Pavement Base Materials. J. Highw. Transp. Res. Dev..

[30] Sheng Y., Li H., Chen S. (2012). Mix Design of Semi-rigid Base Material Based on Anti-erosion Performance. J. Zhengzhou Univ..

[31] Chen W., Liu A., Tang Y., Liu B., Zhang C. (2022). Dissolution and Cavitation of Cement Concrete Pavement Base Under Hydrodynamic Pressure. Concrete.

[32] Gao W., Cui W., Li X. (2018). Experiment and Analysis of Anti-erosion Performance of Semi-rigid Base Surface. J. Highw. Transp. Res. Dev..

[33] (2015). Technical Guidelines for Construction of Highway Roadbases.

[34] (2017). Specifications for Design of Highway Asphalt Pavement.

[35] (2024). Test Methods of Materials Stabilized with Inorganic Binders for Highway Engineering.

[36] Wu H., Jin W., Yan Y., Xia J. (2012). Environmental zonation and life prediction of concrete in frost environments. J. Zhejiang Univ..

[37] Guo Y., Chen X., Wu J., Ji T., Ning Y. (2023). Quantitative visualization monitoring of cracks at shotcrete-rock interface based on acoustic emission. Struct. Control Health Monit..

[38] Liu H., Kuang A., Wang Z., Chu C., Yu H., Lv S. (2023). Investigation on fracture and fatigue performance of cold recycling emulsified asphalt mixture based on acoustic emission parameters. J. Clean. Prod..

[39] Guan H., Pan W., Yang H., Yang Y. (2024). Improvement of the cracking moment-based asphalt mixture splitting test method and splitting strength research. Buildings.

[40] Xiao N., Luo L., Ling F., Tong H. (2022). Numerical study of rock mechanical and fracture property based on CT images. Geomech. Eng..

[41] Yang W., Jin Z., Yang J., Liu Y., Yao J., He Z. (2025). Performance Research on Alkali-Activated Lithium Slag Recycled Cement Stabilized Macadam Mixture Based on Grey Correlation Analysis. J. Chongqing Jiaotong Univ..

